# LRH-1 mediates anti-inflammatory and antifungal phenotype of IL-13-activated macrophages through the PPARγ ligand synthesis

**DOI:** 10.1038/ncomms7801

**Published:** 2015-04-15

**Authors:** Lise Lefèvre, Hélène Authier, Sokrates Stein, Clarisse Majorel, Bettina Couderc, Christophe Dardenne, Mohamad Ala Eddine, Etienne Meunier, José Bernad, Alexis Valentin, Bernard Pipy, Kristina Schoonjans, Agnès Coste

**Affiliations:** 1 UMR MD3, EA2405 Polarisation des Macrophages et Récepteurs Nucléaires dans les Pathologies Inflammatoires et Infectieuses, UPS, Toulouse 31400, France; 2 Université de Toulouse, UMR 152, UPS, Toulouse 31400, France; 3 Metabolic Signaling, Institute of Bioengineering, Ecole Polytechnique Fédérale de Lausanne, Lausanne 1015, Switzerland; 4 EA4553 Individualisation des traitements des cancers ovariens et ORL, UPS, Toulouse 31400, France

## Abstract

Liver receptor homologue-1 (LRH-1) is a nuclear receptor involved in the repression of inflammatory processes in the hepatointestinal tract. Here we report that LRH-1 is expressed in macrophages and induced by the Th2 cytokine IL-13 via a mechanism involving STAT6. We show that loss-of-function of LRH-1 in macrophages impedes IL-13-induced macrophage polarization due to impaired generation of 15-HETE PPARγ ligands. The incapacity to generate 15-HETE metabolites is at least partially caused by the compromised regulation of CYP1A1 and CYP1B1. Mice with LRH-1-deficient macrophages are, furthermore, highly susceptible to gastrointestinal and systemic *Candida albicans* infection. Altogether, these results identify LRH-1 as a critical component of the anti-inflammatory and fungicidal response of alternatively activated macrophages that acts upstream from the IL-13-induced 15-HETE/PPARγ axis.

## Abstract (web summary)

Macrophages activate gene expression of alternative activation program in response to IL-13. Here the authors show that Liver Receptor Homologue-1 regulates synthesis of lipid metabolites stimulating antifungal and repressing proinflammatory genes in macrophages exposed to IL-13 through PPAR activation.

## Graphical abstract

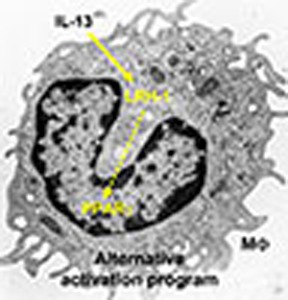

Macrophages orchestrate innate immune responses by initiating and resolving inflammatory signalling programmes. Emerging evidence indicates that the state of macrophage polarization plays a critical role in the regulation of these inflammatory processes. Two different programmes of macrophage activation, the classical (M1) and the alternative differentiation, classify polarized macrophages with either persistence or resolution of inflammation^1^,^2^,^3^. M1 macrophages express high levels of opsonic receptors, involved in the production of pro-inflammatory effector molecules such as reactive oxygen and nitrogen intermediates and pro-inflammatory cytokines (interleukin (IL)-1β, tumour-necrosis factor alpha (TNFα), IL-6 and IL-12). These macrophages contribute to inflammation, microbial killing, regulation of cell proliferation and apoptosis. Alternatively activated macrophages are characterized by abundant levels of the anti-inflammatory cytokine IL-10 and non-opsonic receptors, such as C-type lectin receptors and scavenger receptors (CD36), and resolve inflammation by increasing CD36-mediated efferocytosis and secretion of tissue remodelling/repair mediators^3^,^4^.

The balance of macrophage differentiation in favour of alternatively activated macrophages can be shifted by the activation of the nuclear receptor peroxisome proliferator-activated receptor gamma (PPARγ) (refs 5, 6). PPARγ expression and activity in macrophages is negatively regulated during inflammatory processes^7^,^8^. In addition, activated PPARγ transrepresses many inflammation-activated transcription factors, including nuclear factor-kappaB (NF-κB), signal transducers and activators of transcription (STATs), activator protein 1 (AP1) and nuclear factor of activated T-cells NFAT), resulting in pro-inflammatory mediator inhibition^9^. PPARγ is activated by endogenous ligands derived from the metabolism of arachidonic acid (AA)^9^. Among these ligands, 15-deoxy-Δ^12,14^PGJ_2_ (15d-PGJ2), metabolized through the COX1/COX2 cyclooxygenases, and the 12- and 15-hydroxyeicosatrienoic acids (HETEs), metabolized through 5 and 12/15 lipoxygenases, are essential for PPARγ endogenous activation^5^,^10^,^11^. In addition to cyclooxygenases and lipoxygenases, cytochrome P450 (CYP) enzymes are also considered to be critical for the metabolism of AA in epoxy (EETs) and in hydroxy (HETEs) derivatives^10^,^11^. Within the CYP family, the CYP1 family is mainly involved in the generation of 12- and 15-HETEs through CYP1A1 and CYP1B1 (refs 12, 13).

Liver receptor homologue-1 (LRH-1, NR5A2) is a nuclear receptor highly expressed in the intestine, liver, pancreas and ovary^14^,^15^. Although LRH-1 has been recognized as an orphan receptor, phospholipids, including the phosphatidyl inositol second messengers, and more recently the 12C-fatty acyl-containing phospholipid, dilauroyl phosphatidylcholine (DLPC), have been described to bind the ligand-binding pocket and to act as LRH-1 agonists^16^,^17^,^18^. LRH-1 plays important roles in embryonic development, cholesterol and bile acid homeostasis^14^,^15^ and promotes hepatic glucose sensing through the regulation of the glucokinase enzyme^19^. Several lines of evidence also support a role for LRH-1 in the control of the inflammatory response. While pro-inflammatory factors such as TNFα and lipopolysaccharide (LPS) decrease LRH-1 expression in murine models of human colon tumorigenesis, deficiency of LRH-1 in the intestinal epithelium predisposes mice to intestinal inflammation as a result of a defect in local glucocorticoid production. In the colon from patients with inflammatory bowel disease, inflammation is inversely correlated with the expression of LRH-1 (refs 20, 21). In line with these reports, Venteclef *et al.*^22^,^23^ identified a role for LRH-1 in the negative modulation of the hepatic acute-phase response by inhibiting IL-6- and IL-1β-stimulated haptoglobin, serum amyloid A gene expression in hepatocytes and inducing anti-inflammatory IL-1ra expression. Despite the numerous studies documenting the anti-inflammatory properties of LRH-1 in the liver and gut, no studies so far have focused on the role of LRH-1 in macrophages.

In the present study, we identify LRH-1 as an important regulator of the inflammatory response in macrophages. We demonstrate that LRH-1 is induced by IL-13 via a STAT6-dependent mechanism, which in turn induces the transcriptional activation of CYP1A1 and CYP1B1, two enzymes involved in the generation of 15-HETE PPARγ ligand. Finally, we also demonstrate the importance of intact LRH-1 signalling in the anti-inflammatory and antifungal functions of alternatively activated macrophages, indicating that modulators of LRH-1 activity may have therapeutic potential to restrain infectious and inflammatory diseases.

## Results

### IL-13-mediated LRH-1 gene expression is dependent on STAT6

**Figure 1:**
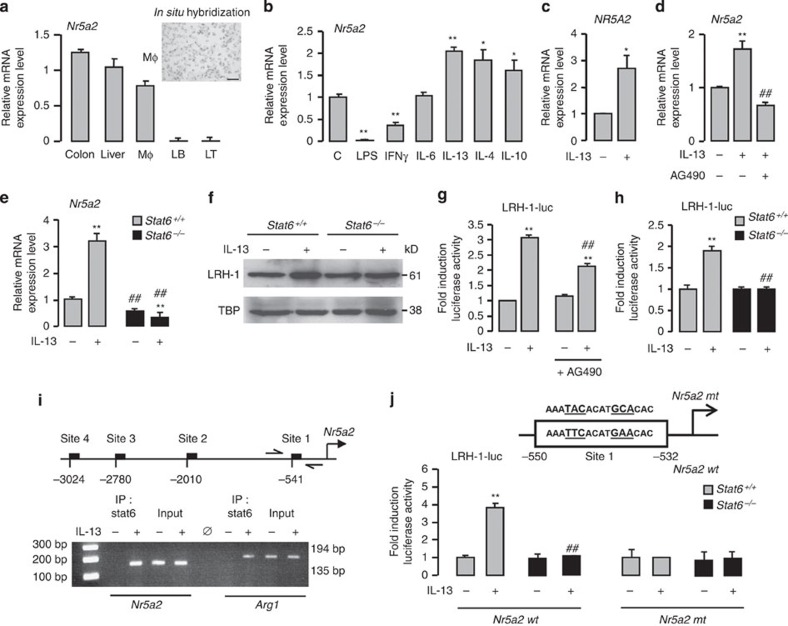
IL-13-mediated LRH-1 gene expression is dependent on STAT6. (**a**) *Nr5a2* mRNA expression in the colon, liver, peritoneal macrophages (MΦ), B (LB) and T (LT) lymphocytes from C57BL/6 mice determined using RT–PCR. Inset shows *in situ* hybridization of *Nr5a2* mRNA in peritoneal macrophages from C57BL/6 mice (scale bar, 25 μm). (**b**,**c**) *Nr5a2* mRNA expression in macrophages from C57BL/6 mice (**b**) and in human macrophages (**c**) treated with the indicated cytokines for 4 h, determined using RT–PCR. The results were represented in fold induction relative to the untreated control or wild-type littermate. (**d**,**e**) *Nr5a2* mRNA expression in macrophages from C57BL/6 mice pretreated with AG490 and stimulated with IL-13 (**d**) and in macrophages from *Stat6*^*+/+*^ and *Stat6*^*−/−*^ mice stimulated with IL-13 for 4 h (**e**), determined using RT–PCR. The results were represented in fold induction relative to the untreated control or wild-type littermate. (**f**) Immunoblot analysis of the nuclear expression of LRH-1 and TBP (Tata-binding protein) in macrophages from *Stat6*^*+/+*^ and *Stat6*^*−/−*^ mice stimulated with IL-13 for 24 h. (**g**,**h**) Luciferase activity in macrophages from C57BL/6 mice transfected with LRH-1 (LRH-1-luc) promoter construct pretreated with AG490 (**g**) or from *Stat6*^*+/+*^ and *Stat6*^*−/−*^ mice (**h**), and treated with IL-13 for 24 h. The results were represented in fold induction relative to the untreated control or wild-type littermate. (**i**) Schematic presentation of the four putative STAT6 response elements in the mouse *Nr5a2* promoter identified by Genomatix algorithm and assessment of STAT6 recruitment to site 1 and to the *Arg1* promoter determined with the ChIP analysis using genomic DNA from C57BL/6 macrophages treated with IL-13 for 4 h. (**j**) Luciferase activity in macrophages from *Stat6*^*+/+*^ and *Stat6*^*−/−*^ mice transfected with LRH-1 promoter constructs and treated with IL-13 for 18 h. The results were represented in fold induction relative to the untreated control. Results correspond to mean±s.e.m. of triplicates. Data are representative of three independent experiments. *P<0.05 **P<0.01 compared with the respective untreated control and ^♯^*P*<0.05, ^♯♯^*P*<0.01 compared with IL-13-treated wild-type littermate. *P* values were determined using Bonferroni–Dunnett method.

The anti-inflammatory properties of LRH-1 are well established in the liver and gut^24^. To elucidate whether LRH-1 also participates in regulating the inflammatory response in macrophages, gene expression profiling was performed. *In situ* hybridization and reverse transcriptase–quantitative PCR (RT–qPCR) revealed that LRH-1 (encoded by the *Nr5a2* gene), known to be expressed in the colon and liver, is also expressed in macrophages but not in B and T immune cells (Fig. 1a). Consistent with the gene expression data, LRH-1 protein was also detected in macrophages (Supplementary Fig. 1b). We next analysed the impact of pro- and anti-inflammatory factors on *Nr5a2* gene expression in primary macrophages. As depicted in Fig. 1b, pro-inflammatory challenges, such LPS and IFNγ exposure, but not IL-6, significantly reduced or abolished *Nr5a2* mRNA expression. Conversely, IL-13, IL-4 and IL-10 cytokines significantly enhanced *Nr5a2* mRNA level in macrophages. Similar to findings in the murine model, *NR5A2* mRNA levels were significantly increased by IL-13 treatment in human monocytes (Fig. 1c). These results suggest that LRH-1 could be part of the transcriptional network mediating alternative activation of macrophages. To test this hypothesis, we analysed the downstream signalling components of IL-13 in more detail (Fig. 1d–i). STAT6, a transcription factor known to be activated by IL-13 is part of the signalling pathway that governs alternative activation^25^. Interestingly, exposure of macrophages with AG490, a Jak-2/STAT6 inhibitor, prevented the IL-13-mediated induction of LRH-1 (Fig. 1d). Consistent with these observations, IL-13 failed to increase *Nr5a2* mRNA and protein levels in macrophages deficient for STAT6 (Fig. 1e,f), suggesting that STAT6 mediates the transcriptional regulation of LRH-1. We then performed transient transfection assays in primary macrophages to assess the effect of IL-13 and STAT6 on *Nr5a2* promoter activity. While 4 h of IL-13 exposure was already sufficient to induce *Nr5a2* promoter activity in wild-type macrophages (Fig. 1g,h), chemical inhibition of STAT6 by AG490 (Fig. 1g) or genetic deletion of STAT6 (Fig. 1h) attenuated or even abolished this response.

To evaluate whether LRH-1 is subject to direct transcriptional control by STAT6, we performed an *in silico* analysis of the *Nr5a2* promoter region. Scanning of the *Nr5a2* promoter sequence for the STAT6 response element (STAT6-RE) canonical motif revealed four putative STAT6-RE (Fig. 1i). Chromatin immunoprecipitation (ChIP) analysis of macrophage DNA from C57BL/6 mice revealed specific recruitment of STAT6 to site 1 at −541, which is most proximal to the transcription initiation site of the gene (Fig. 1i).

To explore the functionality of this site, we next modified by *in vitro* mutagenesis its sequence and we evaluated the mutated *Nr5a2* reporter construct activity on IL-13 exposure (Fig. 1j). Mutation of the STAT6-RE abolished the activity of the Nr5a2 reporter construct in response to IL-13 in *Stat6*^*+/+*^ and *Stat6*^*M−/−*^ macrophages (Fig. 1j). These results demonstrate that STAT6 directly controls the transcription of LRH-1 in response to IL-13.

### LRH-1 is involved in IL-13-induced macrophage activation

In order to assess the role of LRH-1 in IL-13-induced alternative macrophage differentiation, we generated mice in which the *Nr5a2* gene was selectively disrupted in myeloid-derived cells. To generate these animals, mice carrying floxed *Lrh-1* alleles were crossed with transgenic mice that express the Cre recombinase under the control of the mouse phagocyte-selective lysozyme promoter^21^,^26^. Compared with control (*Lrh-1*^*M+/+*^) macrophages, LRH-1 mRNA and protein levels were almost undetectable in macrophages derived from the myeloid cell-specific LRH-1-deficient (*Lrh-1*^*M−/−*^) mice (Supplementary Fig. 1a–c). Furthermore, the disruption of LRH-1 could not be detected in other LRH-1-expressing tissues, such as the liver and the colon (Supplementary Fig. 1d,e).

**Figure 2:**
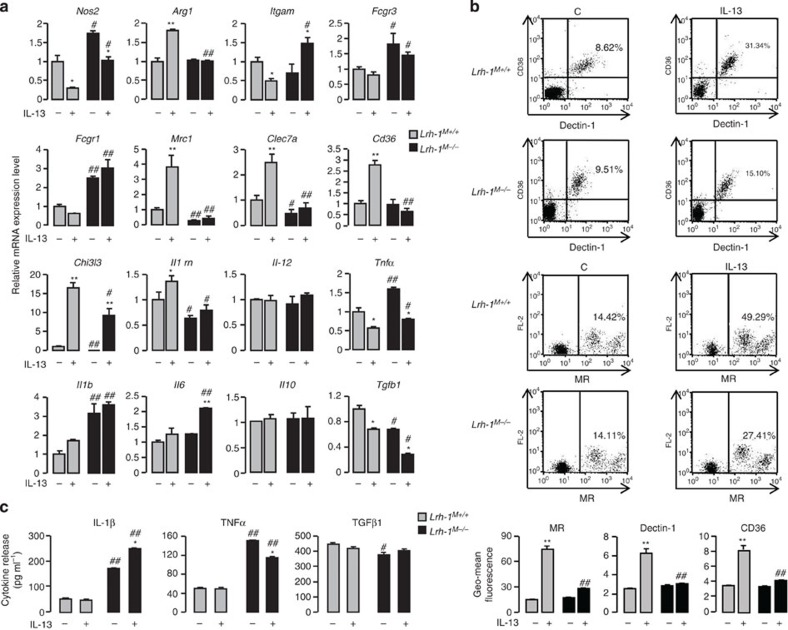
LRH-1 is involved in IL-13-induced alternative activation of macrophages. (**a**) Gene expression analysis of markers of M1 and M2 polarization in peritoneal macrophages from *Lrh-1*^*M+/+*^ and *Lrh-1*^*M−/−*^ mice treated with IL-13 for 4 h, determined using RT–PCR. The results were represented in fold induction relative to the untreated *Lrh-1*^*M+/+*^ littermate. (**b**) Dot-plot representing Dectin-1, CD36 and MR protein expression in macrophages from *Lrh-1*^*M+/+*^ and *Lrh-1*^*M−/−*^ mice treated with IL-13 for 24 h. Numbers indicate the % of positive cells. Graphs represent geomean fluorescence quantification for the indicated proteins. (**c**) Cytokine production of peritoneal macrophages from *Lrh-1*^*M+/+*^ and *Lrh-1*^*M−/−*^ mice after IL-13 treatment and *C. albicans* challenge for 8 h (ratio: 1 macrophage:3 yeasts), quantified by enzyme-linked immunosorbent assay. Results correspond to mean±s.e.m. of triplicates. Data are representative of three independent experiments. *P<0.05, **P<0.01 compared to the respective untreated control and ^♯^*P*<0.05, ^♯♯^*P*<0.01 compared with *Lrh-1*^*M+/+*^+IL-13. *P* values were determined using the Bonferroni–Dunnett method.

We then evaluated the expression of specific markers of classical and alternative activation in untreated or IL-13-treated *Lrh-1*^*M+/+*^ and *Lrh-1*^*M−/−*^ macrophages during 4 h. Overall, *Lrh-1*^*M−/−*^ macrophages displayed an upregulation of M1 markers such as *Nos2* (encoding the inducible nitric oxide synthase) and the Fcγ-receptors *Fcgr3* and *Fcgr*1 (encoding CD16 and CD64 proteins, respectively), which was mirrored by a downregulation of *Chi3l3* (YM1), *Mrc1* (MR), *Clec7a* (Dectin-1), *Il1rn* (IL-1ra) and *Tgfb1* (transforming growth factor (TGF)-β1) alternative activation markers (Fig. 2a,b). This was accompanied by an increase in the mRNA and protein levels of the inflammatory cytokines TNFα, IL-1β and IL-6 (encoded by *Tnfa, Il1b* and *Il-6* genes, respectively; Fig. 2a,c). *Il12* pro-inflammatory and *Il10* anti-inflammatory cytokine mRNA levels remained unchanged in *Lrh-1*^*M+/+*^ and *Lrh-1*^*M−/−*^ macrophages (Fig. 2a). Furthermore, the induction of MR, Dectin-1, CD36, *Arg1* (encoding the arginase 1), *Chi3l3* and *Il1rn* expression by IL-13 was strongly diminished in *Lrh-1*^*M−/−*^ macrophages (Fig. 2a,b). Consistent with reduced alternative activation markers in *Lrh-1*^*M−/−*^ macrophages, the M1 markers such as *Nos2, Itgam* (CD11b), *Fcgr3*, *Fcgr1*, *Il1b* and *Il-6* still remained highly expressed (Fig. 2a–c). Consistent with these findings, the induction of alternative activation gene markers observed after 4 h of IL-13 treatment was amplified after 24 h of IL-13 treatment in *Lrh-1*^*M+/+*^ macrophages (Supplementary Fig. 2a). Moreover, the decrease in alternative activation markers in *Lrh-1*^*M−/−*^ macrophages after 4 h of IL-13 treatment was sustained after 24 h of stimulation (Supplementary Fig. 2a). Altogether, these data indicate that LRH-1 is required for repression of pro-inflammatory state and for optimal induction of alternative macrophage activation by IL-13. These findings are consistent with the robust induction of *Il10*, *Tgfb1*, *Il1rn*, *Mrc1, Clec7a* and *Cd36* gene expression in *Lrh-1*^*M+/+*^ macrophages treated with the LRH-1 agonist DLPC (Supplementary Fig. 2b).

### LRH-1 activates 15-HETE secretion *via* the control of CYP1s

**Figure 3:**
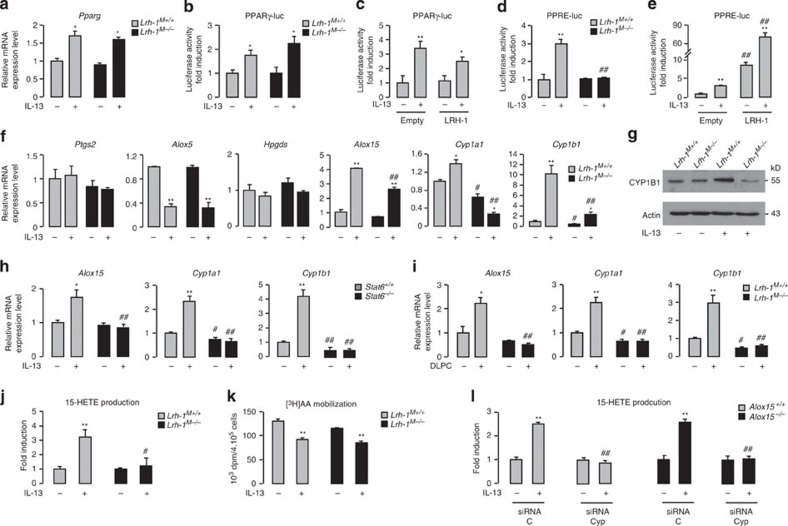
LRH-1 activates CYP1A1- and CYP1B1-dependent 15-HETE production. (**a**) *Pparg* mRNA expression in macrophages from *Lrh-1*^*M+/+*^ and *Lrh-1*^*M−/−*^ mice treated with IL-13 for 4 h, determined using RT–PCR. The results were represented in fold induction relative to the untreated wild-type littermate. (**b**) Luciferase activity in macrophages from *Lrh-1*^*M+/+*^ and *Lrh-1*^*M−/−*^ mice transfected with PPARγ (PPARγ-luc) promoter construct and treated with IL-13 for 4 h. The results were represented in fold induction relative to the respective control. (**c**) Luciferase activity in macrophages from C57BL/6 mice co-transfected with PPARγ (PPARγ-luc) promoter construct in presence (LRH-1) or absence (empty) of LRH-1 (pCMX-LRH-1) and treated with IL-13 for 4 h. The results were represented in fold induction relative to the untreated control (empty). (**d**) Luciferase activity in macrophages from *Lrh-1*^*M+/+*^ and *Lrh-1*^*M−/−*^ mice transfected with a PPRE (PPRE-luc) construct treated with IL-13 for 24 h. The results were represented in fold induction relative to the respective untreated control. (**e**) Luciferase activity of macrophages from C57BL/6 macrophages co-transfected with PPRE (PPRE-luc) construct in presence (LRH-1) or absence (empty) of LRH-1 (pCMX-LRH-1), treated with IL-13 for 24 h. The results were represented in fold induction relative to the respective control. (**f**) Gene expression analysis of arachidonic acid metabolic enzymes in macrophages from *Lrh-1*^*M+/+*^ and *Lrh-1*^*M−/−*^ mice treated with IL-13 for 4 h, determined using RT–PCR. The results were represented in fold induction relative to untreated *Lrh-1*^*M+/+*^. (**g**) Immunoblot analysis of Cyp1b1 and Actin in macrophages from *Lrh-1*^*M+/+*^ and *Lrh-1*^*M−/−*^ mice stimulated with IL-13 for 24 h. (**h**,**i**) Gene expression analysis of *Alox15*, *Cyp1a1* and *Cyp1b1* in macrophages from *Stat6*^*+/+*^ and *Stat6*^*−/−*^ mice treated with IL-13 (**h**) and in macrophages from *Lrh-1*^*M−/−*^ and *Lrh-1*^*M+/+*^ mice stimulated with DLPC for 4 h (**i**), determined using RT–PCR. The results were represented in fold induction relative to untreated wild-type littermate. (**j**) 15-HETE production by macrophages from *Lrh-1*^*M−/−*^ and *Lrh-1*^*M+/+*^ mice stimulated with or without IL-13 quantified by enzyme immunoassay (EIA). The results were represented in fold induction relative to untreated *Lrh-1*^*M+/+*^. (**k**) ^[3H]^AA mobilization in membrane phospholipids of macrophages from *Lrh-1*^*M−/−*^ and *Lrh-1*^*M+/+*^ mice stimulated with IL-13 for 2 h. (**l**) 15-HETE production by macrophages from *Alox15*^*−/−*^ and *Alox15*^*+/+*^ mice stimulated with IL-13 for 24 h and silenced or not for Cyp1a1 and Cyp1b1 (siRNA Cyp) measured by EIA. The results were represented in fold induction relative to respective untreated control (siRNA C). Results correspond to the mean±s.e.m. of triplicates. Data are representative of three independent experiments. *P<0.05, **P<0.01 compared with the respective untreated control and ^♯^*P*<0.05, ^♯♯^*P*<0.01 compared with the corresponding treated or untreated wild-type littermate. *P* values were determined using Bonferroni–Dunnett method.

The nuclear receptor PPARγ is a key component of the signalling pathway triggered by IL-13 and directly controls the expression of markers of alternative activation. To establish whether the increase in alternative activation markers by IL-13 results from direct regulation of PPARγ transcription by LRH-1, we first evaluated *Pparg* mRNA levels in *Lrh-1*^*M+/+*^ and *Lrh-1*^*M−/−*^ macrophages under basal conditions and after IL-13 exposure. The increased *Pparg* mRNA level by IL-13 in *Lrh-1*^*M+/+*^ macrophages was not affected in *Lrh-1*^*M−/−*^ macrophages (Fig. 3a). Moreover, in transient transfection studies, absence of LRH-1 in *Lrh-1*^*M−/−*^ macrophages (Fig. 3b) or conversely ectopic expression of LRH-1 in wild-type macrophages (Fig. 3c) did not significantly affect IL-13-mediated PPARγ promoter induction, further indicating that LRH-1 does not regulate the transcription rate of PPARγ. Next, we examined whether LRH-1 was required for PPARγ activation by assessing the impact of IL-13 on a heterologous PPARγ reporter transfected in *Lrh-1*^*M+/+*^ and *Lrh-1*^*M−/−*^ macrophages. Remarkably, while in *Lrh-1*^*M+/+*^ macrophages IL-13 significantly induced the PPRE luciferase reporter, no such response could be observed in *Lrh-1*^*M−/−*^ macrophages (Fig. 3d). Conversely, co-transfection of the PPRE luciferase reporter with an expression vector for LRH-1 robustly increased PPARγ activation (Fig. 3e), suggesting that LRH-1 induces the activity of PPARγ.

PPARγ is activated by endogenous ligands derived from the metabolism of AA. The COX1/COX2 cyclooxygenases, 5 and 12/15 lipoxygenases and CYP enzymes are considered to be critical for the conversion of AA into endogenous PPARγ ligands. To identify how LRH-1 may have an impact on PPARγ activation, we next explored whether LRH-1 can coordinate PPARγ ligand availability through the control of the expression of these enzymes. The mRNA levels of *Ptgs2* (cyclooxygenase 2), *Alox5* (5 lipoxygenase) and *Hpgds* (prostaglandin-D synthase) after IL-13 stimulation were not differentially expressed in *Lrh-1*^*M+/+*^ and *Lrh-1*^*M−/−*^ macrophages (Fig. 3f). However, IL-13 robustly induced *Alox15* (12/15 lipoxygenase), *Cyp1a1* and *Cyp1b1* gene expression in *Lrh-1*^*M+/+*^ macrophages, while this induction was blunted in *Lrh-1*^*M−/−*^ macrophages. Moreover, Cyp1b1 protein levels were only induced in *Lrh-1*^*M+/+*^ macrophages on IL-13 exposure, but not in *Lrh-1*^*M−/−*^ macrophages (Fig. 3g). Unlike *Cyp1a1* and *Cyp1b1* mRNA levels, which were unresponsive to the IL-13 treatment in *Lrh-1*^*M−/−*^ macrophages, *Alox15* expression was still moderately induced (Fig. 3f), indicating that *Alox15* is only partially controlled by LRH-1.

Consistent with these findings, a strong decrease in *Alox15*, *Cyp1a1* and *Cyp1b1* expression could be observed in both untreated and IL-13-treated *Stat6*^*−/−*^ macrophages (Fig. 3h), further supporting the importance of STAT6 in the regulation of these genes.

To further explore whether STAT6 controls the expression of *Alox15*, *Cyp1a1* and *Cyp1b1* directly or indirectly through the induction of LRH-1, we performed an *in silico* analysis of *Alox15*, *Cyp1a1* and *Cyp1b1* promoters (Supplementary Fig. 3a). This analysis revealed one putative LRH-1 and two putative STAT6-RE in the *Alox15* promoter, with more than 95% of similarity to the consensus REs. Scanning of the *Cyp1a1* and *Cyp1b1* promoter sequences indicated the presence of conserved LRH-1 REs in both promoters, while no conserved STAT6 REs (matrix similarity <0.8) could be identified in these regulatory regions (Supplementary Fig. 3a). Consistent with these findings, DLPC treatment increased *Alox15, Cyp1a1* and *Cyp1b1* gene expression in *Lrh-1*^*M+/+*^ macrophages, but not in *Lrh-1*^*M−/−*^ macrophages (Fig. 3i). These data confirm the importance of LRH-1 in the regulation of *Alox15, Cyp1a1* and *Cyp1b1*.

Finally, to assess whether these effects on gene expression also translate into changes in endogenous ligand availability, 15-HETE production was assessed. Interestingly, while IL-13 exposure robustly enhanced 15-HETE levels in *Lrh-1*^*M+/+*^ macrophages, this effect was completely lost in *Lrh-1*^*M−/−*^ macrophages (Fig. 3j). These findings indicate that LRH-1 is critically required for IL-13-induced 15-HETE production in macrophages. Importantly, IL-13-induced mobilization of AA was similar in *Lrh-1*^*M+/+*^ and *Lrh-1*^*M−/−*^ macrophages (Fig. 3k), indicating that the generation of 15-HETE metabolites through LRH-1 is dependent on AA metabolism.

To further dissect how LRH-1 promotes the production of 15-HETEs in response to IL-13, we assessed 15-HETE production in *Alox15-deficient* macrophages on *Cyp1a1* and *Cyp1b1* short interfering RNA (siRNA)-mediated silencing (Fig. 3l and Supplementary Fig. 3b). Interestingly, the increased 15-HETE production by IL-13 was still conserved in *Alox15*^*−/−*^ macrophages. Furthermore, the simultaneous gene silencing for *Cyp1a1* and *Cyp1b1* in both *Alox15*^*+/+*^ *and Alox15*^*−/−*^ macrophages abolished this induction (Fig. 3l). Altogether, these data indicate that LRH-1 drives the generation of 15-HETE metabolites through its impact on CYP1 gene expression.

**Figure 4:**
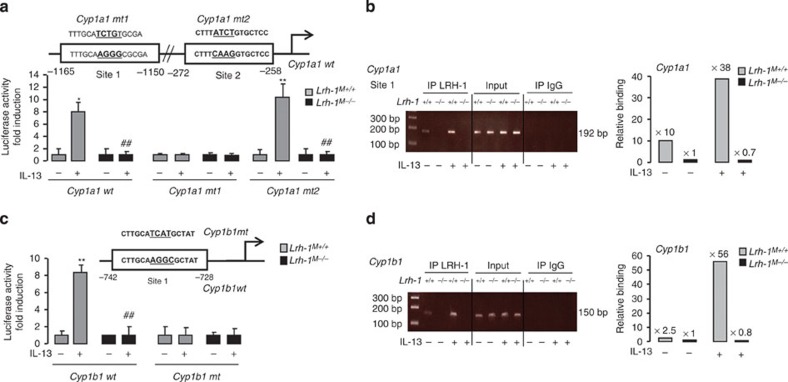
LRH-1 controls the transcription of *Cyp1a1* and *Cyp1b1* genes in response to IL-13. (**a**–**c**) Luciferase activity in macrophages from *Lrh-1*^*M+/+*^ and *Lrh-1*^*M−/−*^ mice transfected with Cyp1a1 promoter constructs (**a**) or Cyp1b1 promoter constructs (**c**) and treated with IL-13 for 18 h. The results were represented in fold induction relative to the untreated control. (**b**,**d**) Assessment of LRH-1 recruitment to site 1 to the *Cyp1a1* promoter (**b**) or *Cyp1b1* promoter (**d**) determined with the ChIP analysis using genomic DNA from *Lrh-1*^*M+/+*^ and *Lrh-1*^*M−/−*^ macrophages treated with IL-13 for 18 h. Results correspond to mean±s.e.m. of triplicates. Data are representative of three independent experiments. *P<0.05, **P<0.01 compared with the respective untreated control and ^♯^*P*<0.05, ^♯♯^*P*<0.01 compared with the corresponding treated wild-type littermate. *P* values were determined using the Bonferroni–Dunnett method.

To define whether *Cyp1a1* and *Cyp1b1* are direct transcriptional targets of LRH-1, transfection assays in *Lrh-1*^*M+/+*^ and *Lrh-1*^*M−/−*^ macrophages were performed using a luciferase reporter containing ±1.2 kb of the promoter of the *Cyp1a1* and *Cyp1b1* genes. IL-13 exposure of *Lrh-1*^*M+/+*^ macrophages resulted in an eightfold increase in reporter activity of both *Cyp1a1* and *Cyp1b* promoters (Fig. 4a,c). Interestingly, genetic deletion of LRH-1 abolished this response, demonstrating that *Cyp1a1* and *Cyp1b1* promoters are directly activated by LRH-1.

To identify the critical LRH-1 REs in the *Cyp1a1* and *Cyp1b1* promoters, we mutagenized the putative RE that were found by in *silico* analysis (Supplementary Fig. 3a), and their response to LRH-1 on IL-13 exposure was compared (Fig. 4a–c). For the *Cyp1a1* promoter, mutation of the first LRH-1 RE (site 1) abolished the activity of the reporter construct in response to IL-13, whereas mutation of site 2 was still responsive in *Lrh-1*^*M+/+*^ macrophages (Fig. 4a). Furthermore, whole inhibition of mutated reporter construct activities in *Lrh-1*^*M−/−*^ macrophages established that site 1 is the principal site transmitting the effect of LRH-1 on the *Cyp1a1* promoter. Thus, this result identified specific recruitment of LRH-1 to site 1, which is most distal to the transcription initiation site in the *Cyp1a1* promoter.

For the *Cyp1b1* promoter, IL-13 treatment failed to increase the activity of the mutated *Cyp1b1* reporter in both *Lrh-1*^*M+/+*^ or *Lrh-1*^*M−/−*^ macrophages (Fig. 4c), indicating that LRH-1 binds and activates the *Cyp1b1* promoter through a unique sequence between −742 and −728 bp upstream of the transcription initiation site of the gene. Finally, ChIP assays were performed. IL-13 enhanced the recruitment of LRH-1 on both *Cyp1a1* and *Cyp1b1* sites in *Lrh-1*^*M+/+*^ macrophages, but not in *Lrh-1*^*M−/−*^ macrophages (Fig. 4b–d). Altogether, these results demonstrate that LRH-1 directly binds *Cyp1a1* and *Cyp1b1* promoters and hence controls the transcription of *Cyp1a1* and *Cyp1b1* genes in response to IL-13.

### LRH-1/CYP1-dependent 15-HETE release induces PPARγ activation

**Figure 5:**
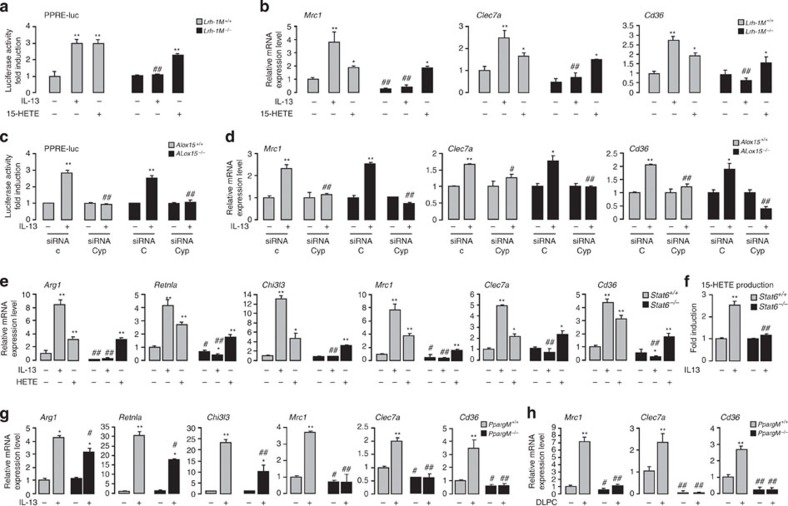
STAT6/LRH-1/PPARγ signaling is required for IL-13-mediated alternative activation of macrophages. (**a**) Luciferase activity in peritoneal macrophages from *Lrh-1*^*M+/+*^ and *Lrh-1*^*M−/−*^ mice transfected with a PPRE (PPRE-luc) construct and treated with IL-13 or 15-HETE for 24 h. (**b**) Gene expression analysis of *Mrc1, Clec7a* and *Cd36* in macrophages from *Lrh-1*^*M+/+*^ and *Lrh-1*^*M−/−*^ mice treated with IL-13 or 15-HETE for 4 h, determined by RT–PCR. (**c**) Luciferase activity of macrophages from *ALox15*^*+/+*^ and *ALox15*^*−/−*^ mice transfected with a PPRE (PPRE-luc) construct and siRNA targeting Cyp1a1 and Cyp1b1 (siRNA Cyp) and treated with IL-13 for 24 h. (**d**) Gene expression analysis of *Mrc1*, *Clec7a* and *Cd36* in macrophages from *ALox15*^*+/+*^ and *ALox15*^*−/−*^ mice transfected with siRNA targeting Cyp1a1 and Cyp1b1 (siRNA Cyp) treated with IL-13 for 4 h and determined by RT-PCR. (**e**,**g**) Gene expression analysis of *Arg1* (arginase 1), *Retnla* (Fizz1), *Chi3l3* (YM1), *Mrc1, Clec7a* and *Cd36* in macrophages from *Stat6*^*+/+*^ and *Stat6*^*−/−*^ mice (**e**) or from *Pparg*^*M+/+*^ and *Pparg*^*M−/−*^ mice (**g**) treated with IL-13 or 15-HETE (**e**) for 24 h, determined by RT-PCR. (**f**) 15-HETE production by macrophages from *Stat6*^*−/−*^ and *Stat6*^*+/+*^ mice stimulated with IL-13 for 24 h measured by EIA. (**h**) Gene expression analysis of *Mrc1, Clec7a* and *Cd36* in macrophages from *Pparg*^*M+/+*^ and *Pparg*^*M−/−*^ treated with DLPC for 4 h, determined by RT-PCR. Results were represented in fold induction compared to the respective untreated control or wild-type littermate and correspond to mean±s.e.m. of triplicates. Data are representative of three independent experiments. *P<0.05, **P<0.01 compared to the respective floxed or not untreated control and ^♯^*P*<0.05, ^♯♯^*P*<0.01 compared to the corresponding untreated or treated wild-type littermate or siRNA control. *P* values were determined using Bonferroni–Dunnett method.

To further determine whether the generation of 15-HETE metabolites through LRH-1 are involved in PPARγ activation, we assessed whether supplementation of 15-HETE can rescue the loss of PPARγ activation in *Lrh-1*^*M−/−*^ macrophages. In contrast to IL-13, which could not induce PPARγ activation in *Lrh-1*^*M−/−*^ macrophages, addition of exogenous 15-HETE efficiently restored the induction of both a PPRE luciferase reporter (Fig. 5a) and of PPARγ target genes such as *Mrc1, Clec7a* and *Cd36* (Fig. 5b) in *Lrh-1*^*M−/−*^ macrophages, indicating that the PPARγ activation through LRH-1 is critically dependent on 15-HETE production.

To confirm that 15-HETE production through the LRH-1/CYP1 axis induces PPARγ activation, we evaluated PPARγ activation in macrophages silenced for *Cyp1a1* and *Cyp1b1*. Interestingly, PPARγ activity as determined by the induction of a PPRE luciferase reporter (Fig. 5c) and the induction of PPARγ target genes (Fig. 5d) by IL-13 were totally inhibited in macrophages deficient for *Cyp1a1* and *Cyp1b1*. Moreover, the induction of a PPRE luciferase reporter (Fig. 5c) and of PPARγ target genes (Fig. 5d) was still significantly enhanced by IL-13 in *Alox15*^*−/−*^ macrophages, showing that the 12/15 lipoxygenase is not required for PPARγ activation mediated by LRH-1. These data are in support of a critical role of CYP1A1 and CYP1B1 in LRH-1-mediated PPARγ activation through 15-HETE synthesis.

### IL-13 activation of macrophages requires STAT6/LRH-1/PPARγ

To determine whether STAT6 controls both directly the transcription of markers of IL-13-mediated alternative activation and indirectly through the activation of the LRH-1/PPARγ axis, we studied the mRNA level of alternative activation markers in STAT6-deficient macrophages. IL-13-augmented induction of *Arg1*, *Chi3l3* (*YM1*)*, Retnla (Fizz1), MR, Clec7a* and *CD36* was detected in *Stat6*^*+/+*^ macrophages but not in *Stat6*^*−/−*^ macrophages (Fig. 5e). The lack of IL-13-augmented induction of alternative markers was associated with a failure of *Stat6*^*−/−*^ macrophages to produce 15-HETE in response to IL-13 (Fig. 5f). Interestingly, the addition of exogenous 15-HETE restored the induction of alternative polarization markers in *Stat6*^*−/−*^ macrophages and not in *Pparγ*^*M−/−*^ macrophages (Fig. 5e and Supplementary Fig. 3c). These data suggest that STAT6 is required for induction of macrophage-alternative activation markers and further support the existence of a PPARγ-dependent mechanism in the regulation of these genes.

Moreover, induction of *Arg1*, *Retnla (Fizz1)* and *Chi3l3* (*YM1*) in response to IL-13 was slightly decreased in *Pparγ*^*M−/−*^ macrophages, whereas the induction of *Mrc1 (MR), Clec7a* and CD36 was completely abrogated in *Pparγ*^*M−/−*^ macrophages (Fig. 5g). These results indicate the existence of distinct regulatory mechanisms involving either STAT6 with a modest contribution of PPARγ or predominantly controlled by the LRH-1/PPARγ axis. In line, the overexpression of *Mrc1*, *Clec7a* and *Cd36* after treatment with DLPC in *Pparγ*^*M+/+*^ macrophages was not detected in *Pparγ*^*M−/−*^ macrophages (Fig. 5h), clearly establishing that LRH-1 acts upstream from PPARγ in the signalling cascade leading to the PPARγ-dependent gene expression.

### IL-13-induced fungicidal properties of macrophages via LRH-1

**Figure 6:**
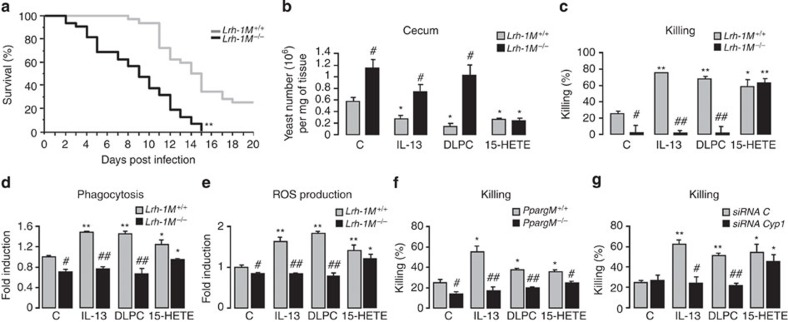
IL-13-induced antifungal properties of macrophages require LRH-1. (**a**) Survival of *Lrh-1*^*M−/−*^ and *Lrh-1*^*M+/+*^ mice to an intraperitoneal injection of *C. albicans* (1.10^8^ yeasts per mouse, *n*=32 per group). Survival (%) was assessed twice daily. *P<0.001 compared with *Lrh-1*^*M+/+*^ mice using log-rank test. (**b**) *Lrh-1*^*M+/+*^ and *Lrh-1*^*M−/−*^ mice were infected with *C. albicans*, and treated i.p. without (C) or with IL-13, DLPC or 15-HETE. *C. albicans* gastrointestinal colonization in the caecum was determined on day 7 using RT–PCR. Data are represented as mean±s.e.m. *P<0.05 compared with the respective untreated control and ^♯^*P*<0.05 compared with the corresponding untreated or treated *Lrh-1*^*M+/+*^. The data are representative of at least two independent experiments (*n*=10 per group). (**c**) Killing assay of *Lrh-1*^*M+/+*^ and *Lrh-1*^*M−/−*^ macrophages incubated with *C. albicans.* (**d**,**e**) Phagocytosis (**d**) and ROS induction (**e**) of *C. albicans* were measured in macrophages from *Lrh-1*^*M−/−*^ and *Lrh-1*^*M+/+*^ mice. Data are expressed as fold induction relative to the fluorescence (**c**) or chemiluminescence (**d**) observed for untreated *Lrh-1*^*M+/+*^. (**f**) Killing assay of *Pparg*^*M+/+*^ and *Pparg*^*M−/−*^ macrophages incubated with *C. albicans.* (**g**) Killing assay of macrophages silenced for Cyp1a1 and Cyp1b1 incubated with *C. albicans.* *P<0.05, **P<0.01 compared with the respective untreated control and ^♯^*P*<0.05 ^♯♯^*P*<0.01 compared with the corresponding treated wild-type littermate or siRNA control. *P* values were determined using Bonferroni–Dunnett method. Results correspond to mean±s.e.m. of triplicates and are representative of at least three independent experiments. For indicated measurements, treatments with IL-13, 15-HETE and DLPC were performed 24 h before the challenge with *C. albicans.*

Previous work from our laboratory established the importance of PPARγ in the fungicidal functions of alternatively activated macrophages^27^. On the basis of the current findings suggesting a role for LRH-1 in PPARγ-mediated alternative polarization following IL-13 stimulation, we next investigated whether deletion of LRH-1 in macrophages could have an impact on the outcome of *Candida albicans* infection. The severe systemic infection of mice with *C. albicans* resulted in a significantly lower survival rate of *Lrh-1*^*M−/−*^ mice compared with *Lrh-1*^*M+/+*^ mice (*P*<0.001; Fig. 6a), supporting a role for LRH-1 in antifungal defence. To further explore the exact function of LRH-1 in the pathophysiology of fungal infection, we evaluated the fungal burden in the intestinal tract and the macrophage microbicidal functions in a murine experimental model of gastrointestinal candidiasis. *Lrh-1*^*M−/−*^ mice infected with *C. albicans* had more severe gastrointestinal infection than their wild-type littermates and showed worsened fungal burden in the caecum (Fig. 6b). Remarkably, IL-13, 15-HETE, as well as DLPC, diminished *C. albicans* gastrointestinal colonization in *Lrh-1*^*M+/+*^ mice. However, these effects were lost in *Lrh-1*^*M−/−*^ mice treated with IL-13 or DLPC, but not when the PPARγ ligand, 15-HETE, was administered to the animals (Fig. 6b).

To investigate whether LRH-1 in macrophages has any relevant microbicidal phenotype, we evaluated the capacity of *Lrh-1*^*M+/+*^ and *Lrh-1*^*M−/−*^ macrophages to kill yeasts *in vitro*. Compared with *Lrh-1*^*M+/+*^ macrophages, *Lrh-1*^*M−/−*^ macrophages showed a defect in their ability to kill *C. albicans,* demonstrating the contribution of LRH-1 in macrophage-intrinsic antifungal activity (Fig. 6c). Consistent with our observation, *Lrh-1*^*M−/−*^ macrophages were less efficient in engulfing *C. albicans* and producing reactive oxygen species (ROS) after fungal challenge (Fig. 6d,e). Moreover, the defect of *Lrh-1*^*M−/−*^ macrophages to exert their antifungal activity was correlated with lower MR and Dectin-1 protein levels after *C. albicans* challenge (Supplementary Fig. 3d). As expected, treatment with IL-13 of *Lrh-1*^*M+/+*^ macrophages increased the killing and the phagocytosis of *C. albicans* and also ROS production in response to *C. albicans*. These inductions were abrogated in *Lrh-1*^*M−/−*^ macrophages, underscoring the importance of LRH-1 in these fungicidal functions (Fig. 6c–e). Similar effects were obtained when macrophages were stimulated with DLPC (Fig. 6c–e). Interestingly, treatment with 15-HETE increased the fungicidal functions in both *Lrh-1*^*M+/+*^ and *Lrh-1*^*M−/−*^ macrophages (Fig. 6c–e). Moreover, treatment with IL-13, DLPC and 15-HETE of *Pparg*^*M−/−*^ macrophages did not increase the killing of *C. albicans* (Fig. 6f), corroborating our findings that PPARγ is downstream from LRH-1 in the signalling pathway triggered by IL-13, leading to macrophage fungicidal activities.

To unequivocally establish that the LRH-1/CYP1/HETE axis is involved in macrophage-intrinsic antifungal activity of IL-13, we evaluated the ability of macrophages silenced for Cyp1a1 and Cyp1b1 (Cyp1) to kill *C. albicans.* Interestingly, the increase in *C. albicans* killing by IL-13 and DLPC was inhibited by the simultaneous gene silencing for Cyp1a1 and Cyp1b1 (Cyp1), but not after 15-HETE stimulation (Fig. 6g). Taken together, these data provide *in vivo* evidence that LRH-1 is involved in the PPARγ-dependent antifungal functions elicited by IL-13 through CYP1-induced 15-HETE production.

## Discussion

The nuclear receptor PPARγ is essential for IL-13-induced alternative differentiation of macrophages^6^,^28^,^29^. We have previously demonstrated that IL-13, via the cPLA_2_ signalling pathway, induced AA mobilization associated with the nuclear localization of 15d-PGJ2, an endogenous PPARγ ligand^5^. Once activated, PPARγ induces the transcription of *Dectin-1*, *MR* and *CD36*, three genes characteristic of the alternative activation^5^,^30^,^31^. Therefore, the processes leading to PPARγ activation, such as AA release and its subsequent metabolic conversion, could be important aspects of alternative polarization because they are limiting factors for PPARγ ligand synthesis.

AA can be metabolized by the COX1/COX2 cyclooxygenases to PGH2, which in turn is transformed by the PGD synthase into 15d-PGJ2 (refs 32, 33). AA can also be directly metabolized to 12- and HETEs, other endogenous PPARγ ligands, through 12/15 lipoxygenases^34^. A third pathway of AA metabolism leading to endogenous PPARγ ligand production is associated with its conversion by the enzymes of the CYP family^35^,^36^,^37^. The CYP enzymes generate two biological and active classes of eicosanoids, the epoxy (EETs) and hydroxy (HETEs) derivatives^10^,^11^. The CYP1 family is mainly involved in the formation of mid-chain HETEs, such as 12- and 15-HETEs, through CYP1A1 and CYP1B1 (refs 12, 13).

Here we report that the nuclear receptor LRH-1 is expressed in macrophages and in response to IL-13 directly binds CYP1A1 and CYP1B1 promoters to positively regulate their transcription. Moreover, 15-HETE production following IL-13 stimulation is impaired in macrophages deficient for LRH-1 and not in macrophages lacking 12/15 lipoxygenase, indicating that LRH-1 drives the generation of 15-HETE metabolites through its impact on CYP1 gene expression. Consistently, our findings showing that the concurrent gene silencing of *Cyp1a1* and *Cyp1b1* in macrophages abolishes the generation of 15-HETE, provide evidence that its production through the LRH-1/CYP1s axis is crucial in PPARγ activation. This is corroborated by the findings that PPARγ activation on IL-13 stimulation is lost in macrophages silenced simultaneously for CYP1A1/CYP1B1 and restored by the addition of exogenous 15-HETE in macrophages lacking LRH-1. Consistent with these observations, treatment of macrophages with the LRH-1 agonist, DLPC, increased the expression of CD36, MR and Dectin-1 PPARγ target genes in wild-type macrophages but not in macrophages lacking PPARγ. Altogether, these results establish that PPARγ activation by IL-13 is dependent on the LRH-1/CYP1/15-HETE pathway. Another endogenous activator to consider in PPARγ activation is 15d-PGJ2. Although we have previously shown that IL-13 generates 15d-PGJ2 production and its nuclear localization in macrophages^5^, the results in this study suggest that it is not sufficient to activate PPARγ. This is supported by previous reports showing that 15d-PGJ2 concentration required to stimulate PPARγ is in the μM range, in contrast to other prostaglandins that are normally active at low nM concentrations^38^,^39^. Thus, the levels generated *in vivo* are not sufficient to be compatible with a role for this metabolite as an endogenous PPARγ ligand^38^,^40^.

Despite the growing knowledge with regard to the biological function of LRH-1, little is known about how LRH-1 is controlled at the transcriptional level. We identified STAT6 as a transcriptional regulator of LRH-1. This was evidenced by the induction of LRH-1 promoter activity by binding of STAT6 to its RE in the LRH-1 promoter and by the decrease in LRH-1 mRNA and protein levels in macrophages lacking STAT6. On the basis of the established role of STAT6 in PPARγ activation and macrophage polarization^41^, these findings identify LRH-1 as a critical component in the signalling cascades that drive PPARγ-mediated alternative macrophage activation. This was further highlighted by the fact that macrophages lacking LRH-1 present an increase in pro-inflammatory cytokines and the simultaneous expression of other M1 markers. The involvement of LRH-1 in anti-inflammatory responses was supported by the robust reduction of LRH-1 gene expression in response to Th1 cytokines and conversely by the upregulation by Th2 cytokines. Interestingly, LRH-1 was also induced in human macrophages in response to the Th2 cytokine IL-13 via a mechanism that is most likely also STAT6-dependent, given the presence of several conserved STAT6 REs in the human *LRH-1* promoter (Supplementary Fig. 3a). Our findings may further explain why during Crohn's disease, characterized by a Th1 cytokine profile, mRNA expression levels of LRH-1 are lower than in ulcerative colitis, characterized by a Th2 immune response^21^. Consistent with the anti-inflammatory role of LRH-1, IL-13-induced alternative activation was impaired in macrophages lacking LRH-1. Indeed, on IL-13 treatment, the induction of several signature genes of alternative activation, including *Arginase 1, YM1, IL-1 receptor antagonist (IL-1ra), MR, Dectin-1* and *CD36*, was significantly impaired in macrophages lacking LRH-1. This is in agreement with reports showing that LRH-1 controls the expression of anti-inflammatory IL-1ra and the scavenger receptor class B type I, two markers specific of alternatively activated macrophages^22^,^23^,^42^.

In addition to the key role of LRH-1 in the acquisition of alternative activation of macrophages, this study also provides mechanistic insight into the hierarchy between STAT6, LRH-1 and PPARγ to achieve this phenotype. Our findings showing that loss of induction of alternative activation markers in Stat6^*−/−*^ macrophages can be restored by exogenous 15-HETE support the notion that STAT6 is required for macrophage-alternative activation through PPARγ-dependent mechanism. Moreover, the use of *Pparγ*^*M−/−*^ macrophages provides evidence for the existence of distinct mechanisms in the transcriptional regulation of genes characteristics of alternative activation. Our results demonstrate that the transcriptional regulation of Arginase 1, Fizz 1 and YM1 involves directly STAT6 with a modest contribution of PPARγ and that Dectin-1, MR and CD36 are regulated indirectly by STAT6 through the LRH-1/PPARγ axis. These observations are not only consistent with the requirement of STAT6 to induce the majority of PPARγ target genes^41^ but also with the identification of PPARγ as a positive regulator of alternative activation^6^.

Consistent with the involvement of the LRH-1/PPARγ pathway in inducing MR and Dectin-1 expression during IL-13-mediated alternative activation, loss of LRH-1 and PPARγ in macrophages also severely compromised their capacity to kill, to engulf *C. albicans* and to produce ROS. This is in line with the fact that LRH-1 is upstream from PPARγ in the signalling pathway leading to the induction of MR and Dectin-1, two C-type lectin receptors strongly involved in the antifungal functions of macrophages against *C. albicans*^5^,^27^,^31^. LRH-1 deficiency in myeloid cells also rendered the mice highly susceptible to gastrointestinal and systemic *C. albicans* infection, highlighting LRH-1 of myeloid lineage as a key effector of host fungicidal functions. Although we have not characterized the role of neutrophils in this infectious context, our *in vitro* and *in vivo* results identify LRH-1 as a nuclear receptor indispensable for alternative activation of macrophages and for its associated antifungal functions.

**Figure 7:**
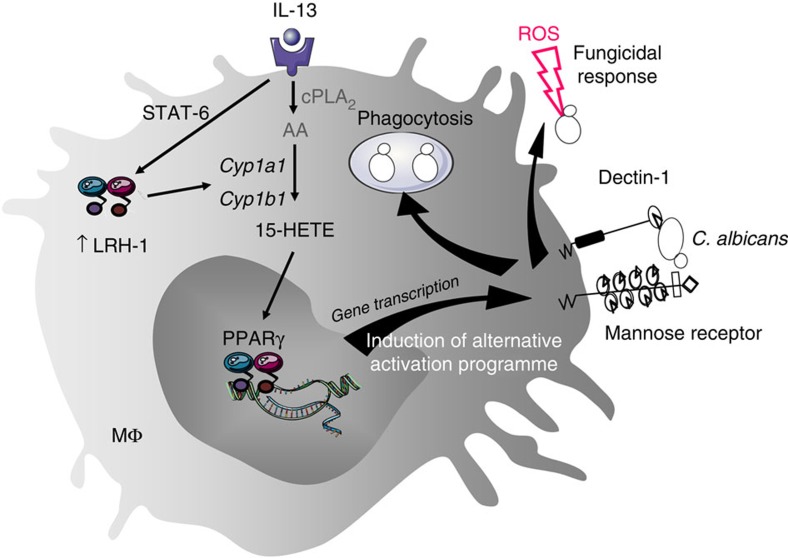
Schematic illustration of the role of LRH-1 in IL-13-alternative activation program of macrophages and in associated fungicidal activities. The alternative polarization of macrophages by IL-13 is dependent on the increase of LRH-1 expression through STAT-6 which controls the expression of Cyp1a1 and Cyp1b1 enzymes leading to the generation of 15-HETE PPARγ ligand.

In conclusion, we have shown that loss of LRH-1 in macrophages prevents IL-13-induced alternative activation of macrophages, demonstrating the pivotal role of LRH-1 in the differentiation of macrophages towards an anti-inflammatory and antifungal phenotype. In response to IL-13, LRH-1 expression is increased in macrophages through STAT6 and controls the expression of CYP1A1 and CYP1B1 enzymes, which catalyses the generation of 15-HETE PPARγ ligand. Altogether, these results establish that the alternative polarization of macrophages by IL-13 is dependent on the STAT6/LRH-1/CYPs/15-HETE/PPARγ axis (Fig. 7). Finally, deletion of LRH-1 in myeloid cells renders mice susceptible to gastrointestinal and systemic *C. albicans* infection, highlighting LRH-1 as a critical factor for antifungal functions. Synthetic agonists of LRH-1 activity may, hence, constitute promising compounds for the treatment of anti-infectious and anti-inflammatory diseases.

## Methods

### Mice

Male mice aged 10–12 weeks on C57BL/6 background were used for *in vitro* and *in vivo* experiments. Mice were bred and handled by following protocols approved by the Conseil Scientifique du Centre de Formation et de Recherche Experimental Médico Chirurgical and the ethics board of the Midi-Pyrénées ethic committee for animal experimentation (Experimentation permit number 31–067, approval no. B3155503). All cages were changed twice weekly, and all manipulations of the animals were carried out in a laminal blow hood under aseptic conditions. The photoperiod was adjusted to 12-h light and 12-h dark. C57BL/6 mice were purchased from Janvier (France) and *Stat6*^*−/−*^ mice and *ALox15*^*−/−*^ mice were purchased from Jackson Laboratories. *Pparg*^*M−/−*^ mice deleted for *Pparg* specifically in macrophages have been described earlier^30^,^43^. *Nr5a2* (encoding LRH-1) macrophage specific knockout mice (referred as *Lrh-1*^*M−/−*^ mice) were obtained by crossing mice carrying floxed *Lrh-1* alleles with transgenic mice expressing the Cre recombinase under the control of the mouse phagocyte-selective lysozyme promoter^21^,^26^. For *Lrh-1*^*M−/−*^ and *Pparg*^*M−/−*^ mice, the corresponding floxed littermates were used as controls throughout all the experiments. Corresponding littermates were used as controls for *Stat6*^*−/−*^ and *ALox15*^*−/−*^ mice.

For the *in vivo* experiments, a gastrointestinal infection with the *C. albicans* strain was established by gavage with 50 × 10^6^ *C. albicans* per mouse (*n*=10 per group). Mice were treated or not intraperitoneally (i.p.) with IL-13 (Clinisciences), DLPC (Sigma) or 15-HETE (Cayman). For IL-13 treatment, injections of 4 μg per mouse were performed 1 day before and 3 days after the infection with *C. albicans* (two injections). For DLPC (300 μg per 10 g of mouse) and 15-HETE (28 μg per 10 g of mouse), i.p. injections were realized 1 day before the day of the infection with *C. albicans* and then every 2 days (five injections). Control groups received saline solution only with DMSO. After 6 days of infection, the ceca were removed aseptically for the experiments.

For *C. albicans* systemic infection, yeasts were administered i.p. (100 × 10^6^yeasts per mouse). Survival studies were conducted using 32 mice per group and were repeated twice.

### Human macrophages

Monocytes were obtained from healthy blood donors (Etablissement Français du Sang, EFS Toulouse). Written informed consents were obtained from the donors under EFS contract no. 21/PVNT/TOU/UPS04/2010–0025. Following articles L1243-4 and R1243-61 of the French Public Health Code, the contract was approved by the French Ministry of Science and Technology (agreement no. AC 2009-921). Human peripheral blood mononuclear cells were isolated from the blood of healthy volunteers by a density gradient centrifugation method on Lymphoprep (Abcys). Monocytes were isolated by adherence to plastic for 2 h in SFM (Gibco) at 37 °C, 5% CO_2_. The macrophages were obtained after 3 days of culture only in SFM medium.

### Preparation of mouse resident peritoneal macrophages

After being killed, resident peritoneal cells were harvested by washing the peritoneal cavity with 5 ml of sterile NaCl 0.9%. Collected cells were centrifuged at 1,500 r.p.m. for 10 min and the cell pellet was suspended in Dulbecco's modified Eagle's medium (Invitrogen) supplemented with glutamine (Invitrogen), penicillin, streptomycin (Invitrogen) and 5% heat-inactivated fetal calf serum. Cells were allowed to adhere for 2 h at 37 °C and 5% CO_2_. Nonadherent cells were then removed by washing with PBS.

### Reverse transcription and real-time PCR

After washing, adherent macrophages were immediately stimulated with IFNγ (40 UI ml^−1^, Clinisciences), IL-6 (50 ng ml^−1^, Clinisciences), LPS (1 ng ml^−1^, Sigma), IL-4 (50 ng ml^−1^, Miltenyi Biotech), IL-13 (50 ng ml^−1^, Clinisciences), IL-10 (50 ng ml^−1^, Clinisciences), 15-HETE (1 μM, Cayman) or DLPC (50 μM, Sigma) for 4 or 24 h. In indicated experiments, adherent macrophages were pre-incubated or not with a Jak-2/STAT6 inhibitor, AG490 (1 nM, Tebu-Bio).

The mRNA preparation was made using the EZ-10 Spin Column Total RNA Minipreps Super Kit (Bio Basic) using the manufacturer's protocol. Synthesis of cDNA was performed according to the manufacturer's recommendations (Thermo electron). RT–qPCR was performed on a LightCycler 480 system using LightCycler SYBR Green I Master (Roche Diagnostics). The primers (Eurogentec) were designed with the software Primer 3. *Actb* (Actin) mRNA was used as the invariant control. Serially diluted samples of pooled cDNA were used as external standards in each run for the quantification. Primer sequences are listed in Supplementary Table 1.

### *In situ* hybridization

*In situ* hybridization was performed with digoxigenin-labelled RNA probe (Plasmid pBSSK Lrh-1) as previously described^44^. Briefly, this manual nonradioactive method allows to detect specific complementary mRNA sequences at the cellular level using digoxigenin-labelled probes in a five-step procedure: hybridization of the probe to pretreated tissue at 65 °C; post-hybridization stringent washes; blocking steps to prepare for the immunodetection; primary antibody anti-DIG-AP incubation; and colorimetric enzymatic detection. The detection step lasts for 2–3 days.

### Western blot analysis

Nuclear protein extracts were prepared, and lysates were subjected to western blotting as described previously^45^. Briefly, nuclear protein lysates were extracted following standard procedures. Protein extracts were separated using SDS–PAGE. After protein transfer, membranes were incubated overnight at 4 °C with either a rabbit anti-Lrh-1 (ref. 21; 1/1,000), a rabbit polyclonal anti-Tbp (Abcam, ab63766, ½,000), a rabbit anti-Cyp1b1 (Santa Cruz, sc-133490, 1/200) or a Actin (Santa Cruz, sc-1615, 1/1,000) and then for 1 h at room temperature with a peroxidase conjugated secondary antibody. Membranes were washed, and proteins were visualized with the SuperSignal West Pico Chemiluminescent Substrate (ThermoScientific). Images have been cropped for presentation. Full-size images are presented in Supplementary Fig. 4.

### Transfection experiments

Macrophages were pre-incubated or not with AG490 (1 nM) and then incubated with 1 μg of DNA per well of the indicated plasmids (pGL3 promoter LRH-1-luciferase, pCMX-LRH-1, PPRE luciferase, pGL3 promoter PPARγ-luciferase, pGL4.12 promoter Cyp1a1-luciferase or pGL4.12 promoter Cyp1b1-luciferase) with JetPei (Polyplus transfection) for 8 h according to the manufacturer's instructions. Then, the cells were stimulated or not with IL-13 (50 ng ml^−1^) for 18 h. Supernatant was removed, luciferase substrate was added and luminescence was measured with the Envision luminometer (Perkin Elmer).

For siRNA experiments, mouse Cyp1a1 and Cyp1b1 and control siRNA were purchased from Origene. Macrophages were incubated with 20 nM of control siRNA or Cyp1a1 and Cyp1b1 siRNA and with Lipofectamine 2000 (Invitrogen) for 18 h according to the manufacturer's instructions. Cells were then stimulated with IL-13, DLPC or 15-HETE for 18 h.

### ChIP

ChIP analysis was performed as described previously with minor adaptations^46^. Briefly, the liver and colon from Lrh-1+/+ and Lrh-1−/− mice were lysed (5 mM PIPES pH 8.0, 85 mM KCl, 0.5% NP40 with protease inhibitors). The pellets or adherent macrophages from indicated mice were cross-linked with 1% formaldehyde for 15 min at room temperature. The cells were then lysed in nuclear lysis buffer (50 mM Tris-HCl pH 8.1, 10 mM EDTA, 1% SDS with protease inhibitors) and sonicated at 30% maximum power eight times. The supernatant was diluted in immunoprecipitation-dilution buffer (0.01% SDS, 1.1% Trition X-100, 1.2 mM EDTA, 16.7 mM Tris-Cl pH 8.1 and 167 mM NaCl with protease inhibitor) and precleared with Protein A agarose/salmon sperm DNA beads (Invitrogen 101141). The samples were immunoprecipitated overnight at 4 °C with a rabbit Lrh-1 antibody^21^, normal rabbit IgG (Santa Cruz, sc-2027, 7 μl/ml) or with a rabbit STAT6 antibody (Santa Cruz, sc-981, 7 μl ml^−1^). The beads were then washed in low-salt buffer (0.1% SDS, 1% Triton X-100, 2 mM EDTA, 20 mM Tris-HCl pH 8 and 150 mM NaCl), high-salt buffer (0.1% SDS, 1% Triton X-100, 2 mM EDTA, 20 mM Tris-HCl pH 8 and 500 mM NaCl) and LiCl buffer (1% NP40, 1% deoxycholate, 1 mM EDTA, 10 mM Tris-HCl pH 8 and 250 mM LiCl). The samples were then boiled in chelex followed by incubation with proteinase K solution (10 μg ml^−1^ proteinase K; 10 mM EDTA; and 37 mM Tris-HCl, pH 6.5) at 55 °C for 30 min. DNA was purified using the EZ-10 Spin Column Total RNA Minipreps Super Kit (Bio Basic), after which qPCR was performed. Data were normalized for GAPDH promoter binding and expressed relative to IgG. ChIP primer sequences are listed in Supplementary Table 2.

### Reporter assays and site-directed mutagenesis

Genomic DNA was extracted from mouse kidneys and the corresponding Cyp1a1 and Cyp1b1 promoter fragments were amplified by PCR with primers containing KpnI and XhoI restriction sites. The PCR products were then cloned into pENTR-D/TOPO (Invitrogen/Lifetechnologies) plasmids, which were digested with KpnI and XhoI and then ligated into pGL4.12 (Promega) reporter plasmids.

Mutagenesis was carried out with the GeneArt mutagenesis kit (Invitrogen/Lifetechnologies) according to the manufacturer's instructions. The sequences of reporter constructs were analysed and confirmed.

### Flow cytometry

The analysis was performed on nonadherent macrophages^47^ harvested by washing the peritoneal cavity with 5 ml of sterile NaCl 0.9%. Collected cells were centrifuged at 1,500 r.p.m. for 10 min and the cell pellet was suspended in PBS medium supplemented with 1% fetal calf serum (FCS). Surface expressed Dectin-1 or CD36 was detected, respectively, using fluoroscein isothiocyanate (FITC)-Dectin-1 monoclonal antibody (mAb; Serotec MCA2289F, 1/100) or PE-CD36 mAb (Santa Cruz, sc-13572, 1/100) and was compared with an irrelevant appropriate isotype control. To evaluate the mannose receptor (MR) surface expression, we have used MR-specific ligand conjugated with FITC (Sigma A7790, 1 mg ml^−1^). All stainings were performed on PBS—1% FCS medium. A population of 10,000 cells was analysed for each data point. All analyses were carried out in a Becton Dickinson FACScalibur using the CellQuestPro software.

B and T lymphocytes were isolated from the mouse spleen with a PE-B220 mAb (RD System FAB1217P, 1/10) and a PE-CD3 mAb (eBiosciences MCA500, 1/10) using a Becton Dickinson Influx cell sorter.

### ELISA Cytokine titration and EIA quantification of 15-HETE

Peritoneal macrophages were stimulated with IL-13 for 18 h and challenged with non-opsonized *C. albicans* at a yeast-to-macrophage ratio of 3:1 for 8 h. The production of TNF-α, IL-1β and TGF-β in the cell supernatants was determined with a commercially available OptiEIA kit (BD Biosciences) according to the manufacturer's instructions.

For 15-HETE quantification, the macrophages were stimulated with IL-13 for 18 h and 15-HETE were measured using EIA as recommended by the manufacturer's protocol (15(S)-HETE EIA kit, Cayman).

### AA mobilization

Peritoneal macrophages were prelabelled with [^3^H]AA (1 μCi per well, Perkin Elmer) for 18 h. The prelabelled macrophages were then treated with IL-13 (50 ng ml^−1^) for 1 h. The cellular lipids were extracted twice with hexane/isopropanol (3:2, v/v) and the [^3^H]AA content in membrane phospholipids was quantified by measurement of the radioactivity by beta liquid scintillation counting, as described with minor adaptations^48^.

### *C. albicans* strain

The strain of *C. albicans* used throughout these experiments was isolated from a blood culture of a Toulouse-Rangueil Hospital patient^5^. Fluorescent *C. albicans* was prepared by adding *C. albicans* to FITC (Sigma) dissolved in sodium carbonate buffer (pH 9.5) at room temperature for 3 h and washed by centrifugation three times in sodium carbonate buffer before storage in aliquots of water at 4 °C.

### Phagocytosis assay and ROS quantification

For analysis of phagocytosis of *C. albicans*, cultured macrophages were pretreated or not with IL-13, 15-HETE or DLPC for 18 h and then challenged with six FITC-labelled yeasts per macrophage. Phagocytosis was initiated at 37 °C with 5% CO_2_ and stopped after 1 h by washing the macrophages with ice-cold PBS. The number of *C. albicans* engulfed by macrophages was determined with fluorescence quantification using the Envision (Perkin Elmer) fluorimetry-based approach.

The oxygen-dependent respiratory burst of macrophages (ROS production) was measured by chemiluminescence in the presence of 5-amino-2,3-dihydro-1,4-phthalazinedione (luminol) using a thermostatically (37 °C) controlled luminometer (Wallac 1420 Victor2). The generation of chemoluminescence was monitored continuously for 1 h after incubation of the cells with luminol (66 μM) and pretreatment with IL-13, 15-HETE or DLPC for 18 h and challenge with *C. albicans* (yeast-to-macrophage ratio: 3:1). Statistical analysis was performed using the area under the curve expressed in counts × seconds.

### Killing assay

The killing assay was performed as previously described^49^. Cells were allowed to interact for 30 min at 37 °C with *C. albicans* (at a ratio of 0.3 yeast per macrophage) and unbound yeasts were removed by four washes with medium. Macrophages were then incubated at 37 °C for 4 h. Control plates were kept at 4 °C to provide a measure of live *C. albicans* in the wells. After incubation, the medium was removed and cells were lysed by incubation for 5 min at 25 °C with water at a pH of 11. An excess of PBS was used to neutralize the lysis buffer, and CFU *C. albicans* was determined by plating on Sabouraud plates and incubation overnight at 37 °C.

### Quantification of *C. albicans* in the caecum

*Cell lysis and DNA extraction*. After mouse infection, ceca were aseptically removed and then crushed using lysing matrix tubes (MP Biomedicals). Tissue sample homogenate (250 μl) was resuspended in 200 μl of lysis buffer for 2 h at 65 °C and DNA was then extracted with isopropanol and eluted with an elution buffer (High Pure PCR Template preparation kit, Roche Diagnostics).

*Light cycler-based PCR assay*. The Light Cycler PCR and detection system (Roche Diagnostics) was used for amplification and online quantification. PCR analysis was performed as described previously^27^. Serially diluted samples of genomic fungal DNA obtained from *C. albicans* cultures (40 × 10^6^ cells) were used as external standards in each run. Cycle numbers of the logarithmic linear phase were plotted against the logarithm of the concentration of template DNA to evaluate the number of yeast cells present in each tissue sample homogenate.

### Statistical analysis

For each experiment, the data were subjected to one-way analysis of variance followed by the means multiple comparison method of Bonferroni–Dunnett. For survival study, statistical significance was determined by a log-rank test. *P*<0.05 was considered as the level of statistical significance.

## Author contributions

A.C., K.S., B.P. and L.L. designed the study, analysed the data and wrote the manuscript. L.L. and A.H. performed and analysed the experiments. S.S., C.M., B.C., C.D., M.A.E., E.M., J.B. and A.V. generated tools and/or helped with specific experiments.

## Additional information

**How to cite this article:** Lefèvre, L. *et al.* LRH-1 mediates anti-inflammatory and antifungal phenotype of IL-13-activated macrophages through the PPARγ ligand synthesis. *Nat. Commun.* 6:6801 doi: 10.1038/ncomms7801 (2015).

## Supplementary Material

Supplementary Information Supplementary Figures 1-4 and Supplementary Tables 1-2
