# Supplementary material for: LRH-1 mediates anti-inflammatory and antifungal phenotype of IL-13-activated macrophages through the PPARγ ligand synthesis
Source: Nat Commun. 2015 Apr 15;6:6801. doi: 10.1038/ncomms7801 (PMC4410638; doi:10.1038/ncomms7801)
Supplement: Supplementary Information — Supplementary Figures 1-4 and Supplementary Tables 1-2 [file ncomms7801-s1.pdf]

Supplementary Figure 1.

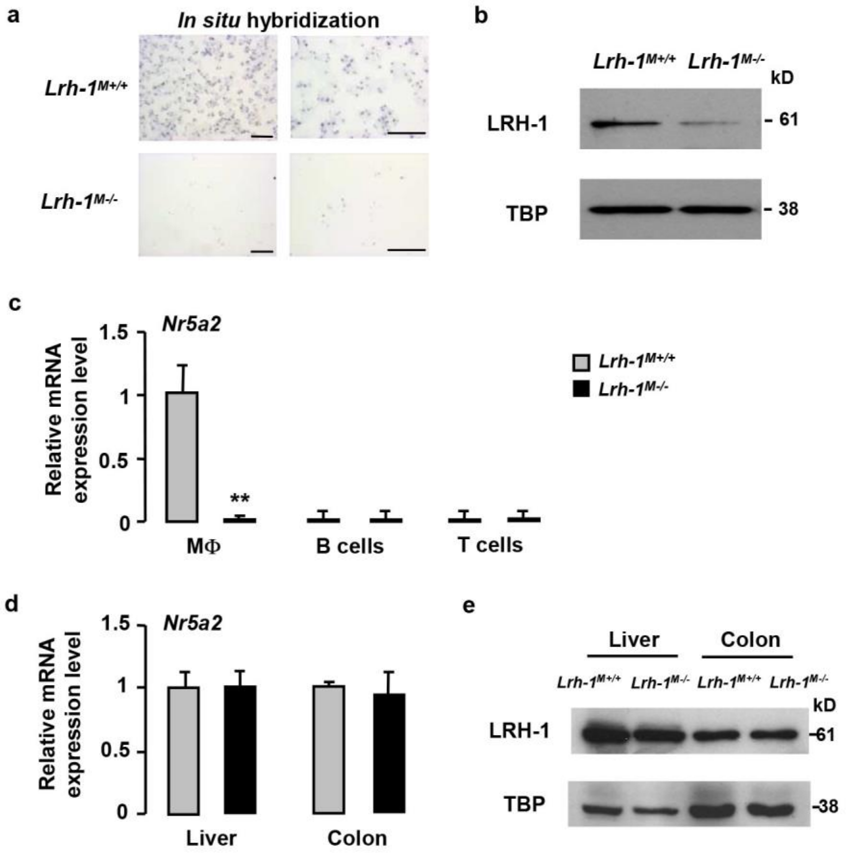

Supplementary Figure 1. Validation of the macrophage-specific *Lrh-1*$^{M-/-}$ mice model.

(a) *In situ* hybridization of *Nr5a2* mRNA in macrophages from *Lrh-1*$^{M+/+}$ (upper panels) or *Lrh-1*$^{M-/-}$ (lower panels) mice.

(b) Immunoblot analysis of nuclear LRH-1 and TBP protein level in macrophages from *Lrh-1*$^{M+/+}$ and *Lrh-1*$^{M-/-}$ mice.

(c-d) Gene expression analysis of *Nr5a2* in macrophages (MΦ), B cells, T cells (c) and in liver and colon (d) of *Lrh-1*$^{M+/+}$ or *Lrh-1*$^{M-/-}$ mice, determined by RT-PCR. **p<0.01 compared to *Lrh-1*$^{M+/+}$. P values were determined using Bonferroni-Dunnett method.

(e) Immunoblot analysis of nuclear LRH-1 and TBP protein level in colon and liver from *Lrh-1*$^{M+/+}$ and *Lrh-1*$^{M-/-}$ mice.

Results correspond to mean ± SEM of triplicates. Data are representative of three independent experiments.

Supplementary Figure 2.

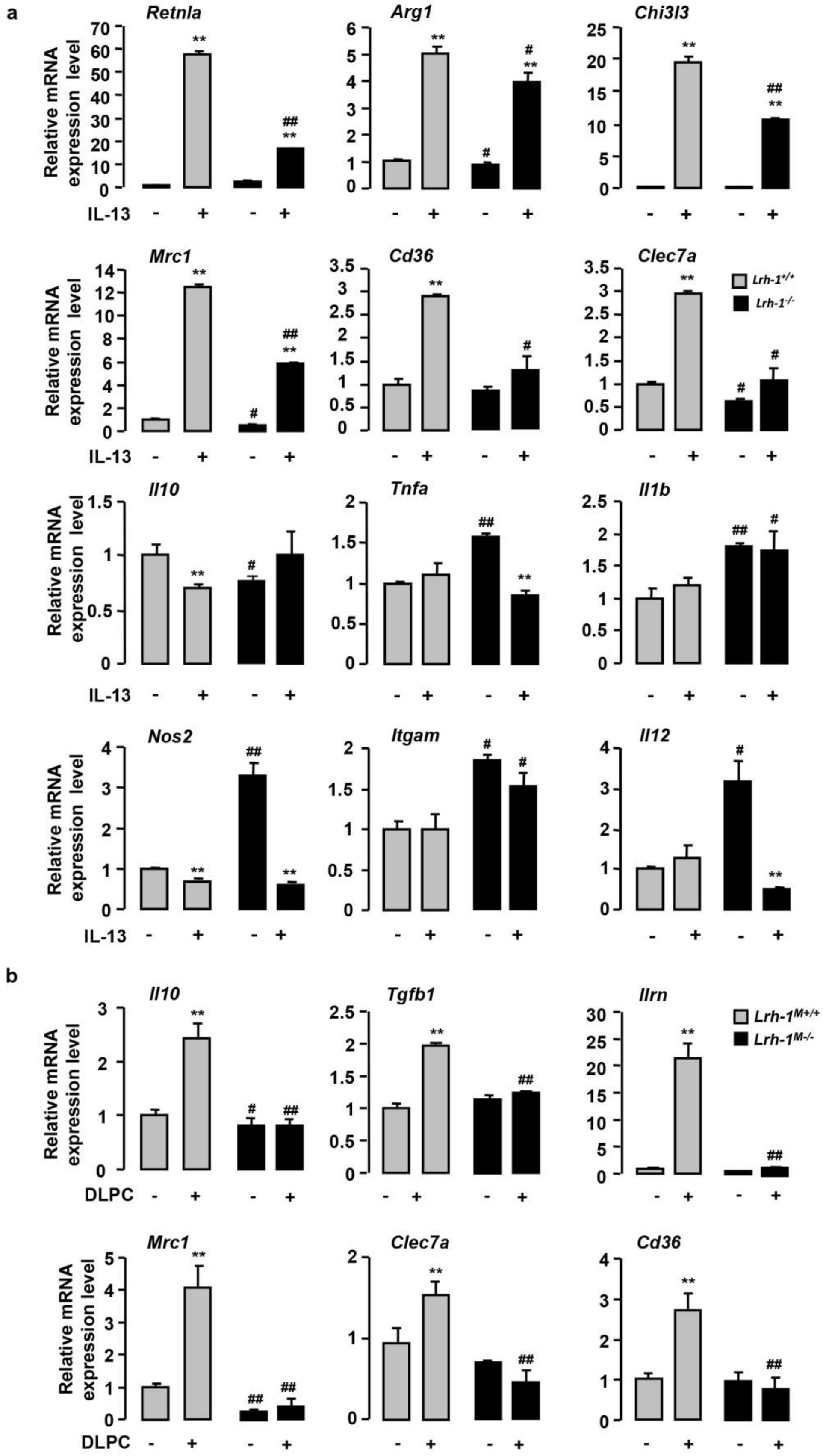

Supplementary Figure 2.

(a) Gene expression analysis of markers of M2 and M1 polarization in peritoneal macrophages from *Lrh-1*$^{M+/+}$ and *Lrh-1*$^{M-/-}$ mice treated with IL-13 for 24h, determined by RT-PCR. The results were represented in fold induction relative to the untreated *Lrh-1*$^{M+/+}$ control.

(b) Gene expression analysis of *Il10*, *Tgfb1*, *Ilrn*, *Mrc1, Clec7a* and *Cd36* in macrophages from *Lrh-1*$^{M+/+}$ and *Lrh-1*$^{M-/-}$ mice treated with DLPC for 4h, determined by RT-PCR. The results were represented in fold induction relative to the untreated *Lrh-1*$^{M+/+}$ control.

Results correspond to mean ± SEM of triplicates. Data are representative of three independent experiments. *p<0.05, **p<0.01 compared to the respective untreated control and $^{\#}$p<0.05, $^{\#\#}$p<0.01 compared to *Lrh-1*$^{M+/+}$ + IL-13. P values were determined using Bonferroni-Dunnett method.

Supplementary Figure 3.

a

Gene	Transcription factor	Upstream position	DNA strand	Matrix similarity	Sequence
Alox15	STAT6	492-510	(+)	0.976	ttgattct**CAGAA**gggaga
		490-508	(-)	0.957	tcc**TTC**tga**GAA**tcaaga
	LRH-1	191-205	(-)	0.954	ggac**CAAGG**g**C**aggt
Cyp1a1	LRH-1	584-598	(-)	0.920	ggtt**CAAGG**c**C**tctg
		258-272	(-)	0.963	cttt**CAAGG**tgctcc
Cyp1b1	LRH-1	728-742	(+)	0.950	cttg**CAAGG**cgctat
NR5A2 human	STAT6	310-328	(+)	0.885	tatt**TTC**Aaaa**GAA**caagt
		109-127	(-)	0.864	taaa**TCCC**tat**GAA**gttaa
		560-578	(-)	0.915	gggg**TT**ggg**GGAA**ggctgg
		115-133	(+)	0.943	tctt**TTC**tc**GGAA**tgttcc

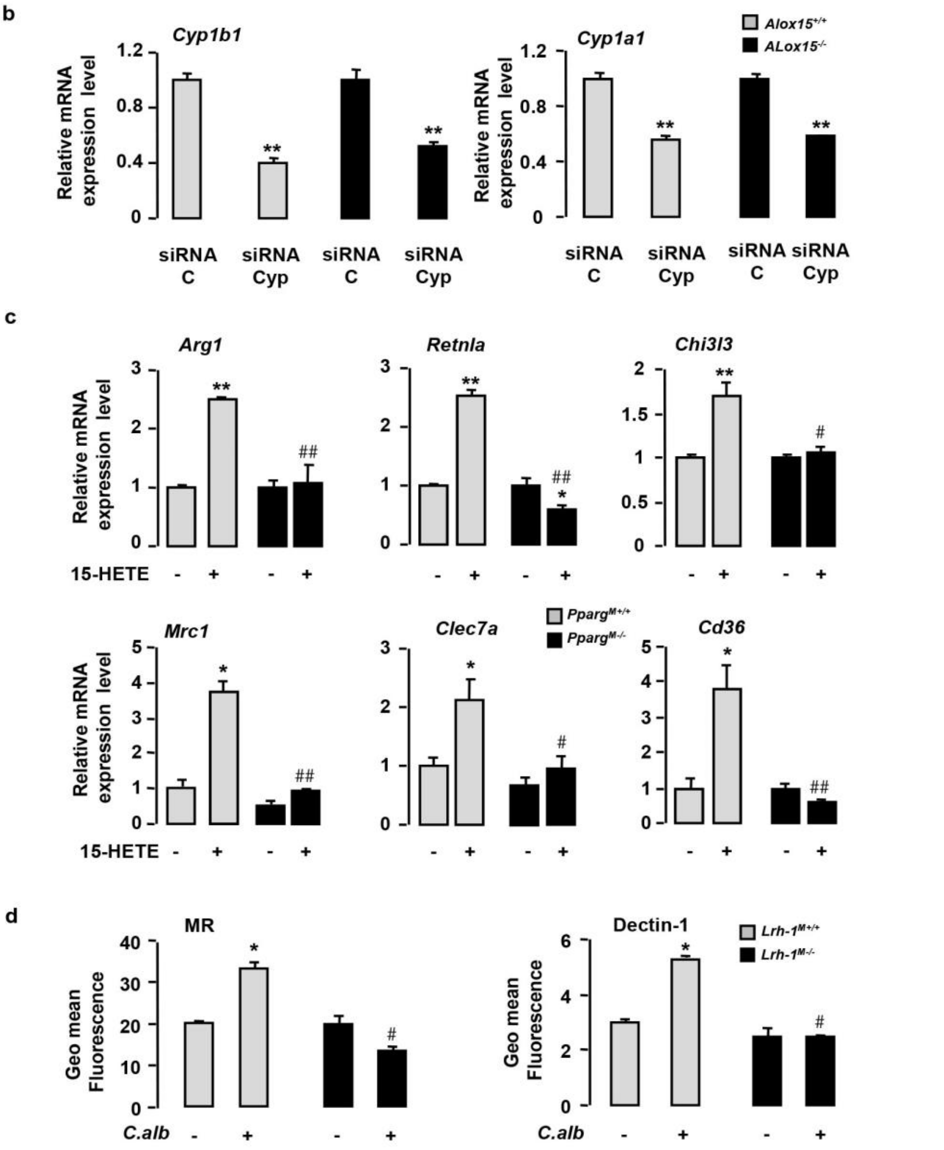

Supplementary Figure 3.

(a) Computational analysis of *Alox15*, *Cyp1a1*, *Cyp1b1* and human *NR5A2* promoters containing putative STAT6 and LRH-1 response elements (bold) identified by Genomatix algorithm.

(b) *Cyp1a1* and *Cyp1b1* mRNA expression on *Alox15*$^{+/+}$ and *Alox15*$^{-/-}$ macrophages transfected with siRNA targeting Cyp1a1 and Cyp1b1 (siRNA Cyp) or control siRNA (siRNA control), determined by RT-PCR.

(c) Gene expression analysis of *Arg1*, *Retnla*, *Chi3l3*, *Mrc1, Clec7a* and *Cd36* in macrophages from *Pparg*$^{M+/+}$ and *Pparg*$^{M-/-}$ mice treated with 15-HETE for 4h, determined by RT-PCR.

(d) MR and Dectin-1 protein expression in macrophages from *Lrh-1*$^{M+/+}$ and *Lrh-1*$^{M-/-}$ mice challenged with *C. albicans* for 24h, quantified by flow cytometry.

Results correspond to mean ± SEM of triplicates. Data are representative of three independent experiments. *p<0.05, **p<0.01 compared to the respective untreated control and $^{\#}$p<0.05, $^{\#\#}$p<0.01 compared to IL-13 stimulated cells. P values were determined using Bonferroni-Dunnett method.

Supplementary Figure 4.

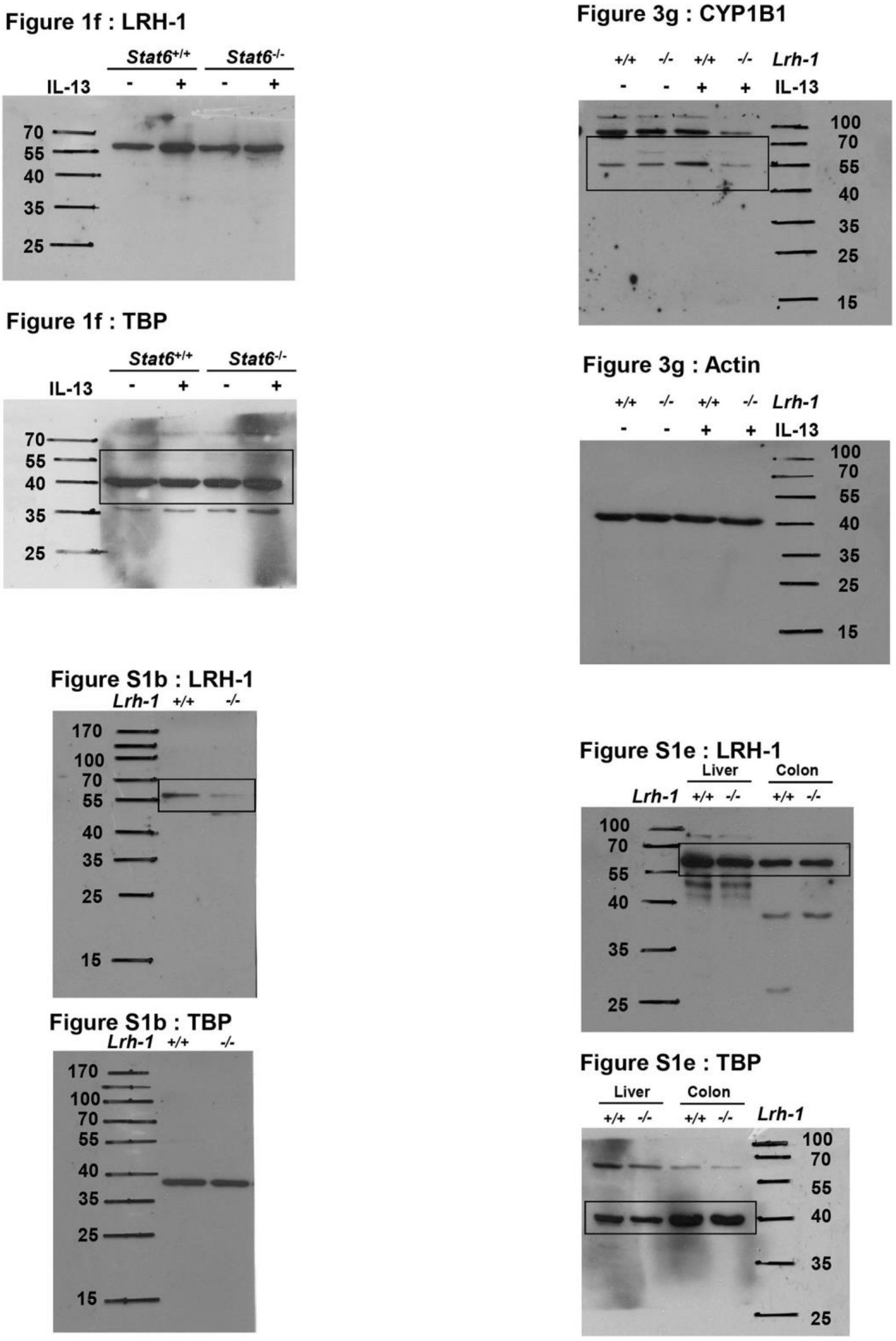

Supplementary Figure 4. Lists of original gel images of western blot analysis. Boxes highlight lanes used in figures.

Supplementary Table 1. Murine and Human primer sequence

Gene	**sequence**
Actb	sense 5’ AGC-CAT-GTA-CGT-AGC-CAT-CC 3’<br>antisense 5’ CTC-TCA-GCT-GTG-GTG-GTG-AA 3’
Chi3l3	sense 5’ AAT-GAT-TCC-TGC-TCC-TGT-GG 3’<br>antisense 5’ ACT-TTG-ATG-GCC-TCA-ACC-TG 3’
Retnla	sense 5’ CTG-GGT-TCT-CCA-CCT-CTT-CA 3’<br>antisense 5’ TGC-TGG-GAT-GAC-TGC-TAC-TG 3’
Fcgr3	sense 5’ TGC-TCC-ATT-TGA-CAC-CGA-TA 3’<br>antisense 5’ TGT-TTG-CTT-TTG-CAG-ACA-GG 3’
Tnfα	sense 5’ AGG-CTG-TGC-ATT-GCA-CCT-CA 3’<br>antisense 5’ GGG-ACA-GTG-ACC-TGC-ACT-GT 3’
Il10	sense 5’ TTT-TCA-CAG-GGG-AGA-AAT-CG 3’<br>antisense 5’ CCA-AGC-CTT-ATC-GGA-AAT-GA 3’
Il1b	sense 5’ GAT-CCA-CAC-TCT-CCA-GCT-GCA 3’<br>antisense 5’ CAA-CCA-ACA-AGT-GAT-ATT-CTC-CAT-G 3’
Il6	sense 5’ AAG-TGC-ATC-ATC-GTT-GTT-CAT-ACA 3’<br>antisense 5’ GAG-GAT-ACC-ACT-CCC-AAC-AGA-CC 3’
Arg1	sense 5’ AGA-GCT-GAC-AGC-AAC-CCT-GT 3’<br>antisense 5’ GGA-TCC-AGA-AGG-TGA-TGG-AA 3’
Fcgr1	Sense 5’ ACC TGT ATT CGT CAC TGT CC 3’<br>Antisense 5’ GTT ATT GCC ACC AAG GCT GT 3’
Nos2	Sense 5’ ACC-ACT-CGT-ACT-TGG-GAT-GT 3’

	Antisense 5'CAC-CTT-GGA-CTT-CAC-CCA-GT 3'
Cd36	sense 5' GCA-GAA-TCA-AGG-GAG-AGC-AC 3'<br>antisense 5' GAG-CAA-CTG-GTG-GAT-GGT-TT 3'
Mrc1	sense 5' ATG-CCA-AGT-GGG-AAA-ATC-TG 3'<br>antisense 5' TGT-AGC-AGT-GGC-CTG-CAT-AG 3'
Clec7a	sense 5' CAT-CGT-CTC-ACC-GTA-TTA-ATG-CAT 3'<br>antisense 5' CCC-AGA-ACC-ATG-GCC-CTT 3'
Il1rn	Sense 5' ACA-TGG-CAA-ACA-ACA-CAG-GA 3'<br>Antisense 5' TAG-CAA-ATG-AGC-CAC-AGA-CG 3'
Tgfb1	Sense 5' AGG-TTG-GCA-TTC-CAC-TTC-AC 3'<br>Antisense 5' AGG-GGC-CTC-TAA-GAG-CAG-TC 3'
Alox15	Sense 5'GAT-TGT-GCC-ATC-CTT-CCA-GT 3'<br>Antisense 5' CAG-GGA-TCG-GAG-TAC-ACG-TT 3'
Alox5	Sense 5' GTG-CTG-CTT-GAG-GAT-GTG-AA 3'<br>Antisense 5'CTA-CGA-TGT-CAC-CGT-GGA-TG 3'
Nr5a2	Sense 5' TCA-TGC-TGC-CCA-AAG-TGG-AGA 3'<br>Antisense 5' TGG-TTT-TGG-ACA-GTT-CGC-TT 3'
Cyp1a1	Sense 5' CAG-GTA-ACG-GAG-GAC-AGG-AA 3'<br>Antisense 5' GGC-CAC-TTT-GAC-CCT-TAC-AA 3'
Cyp1b1	Sense 5' TAA-TGA-AGC-CGT-CCT-TGT-CC 3'<br>Antisense 5' TTC-TCC-AGC-TTT-TTG-CCT-GT 3'
Hpgds	Sense 5' CCA-GCC-CTC-TGA-CTG-ACT-TC 3'

	Antisense 5’ AGT-GGT-GGA-GGC-CAA-CTA-TG 3’
Ptgs2	Sense 5’ GCT-CGG-CTT-CCA-GTA-TTG-AG 3’<br>Antisense 5’ AGA-AGG-AAA-TGG-CTG-CAG-AA 3’
Itgam	Sense 5’ AGA-TCG-TCT-TGG-CAG-ATG-CT 3’<br>Antisense 5’ GAC-TCA-GTG-AGC-CCC-ATC-AT 3’
Pparg	Sense 5’ AAT-CCT-TGG-CCC-TCT-GAG-AT 3’<br>Antisense 5’ TTT-TCA-AGG-GTG-CCA-GTT-TC 3’
Human NR5A2	sense 5’ GAC-CAC-ACC-ATT-CCT-CTC-CA 3’<br>antisense 5’ TTA-GGT-AAG-CTG-GGC-GTT-GA 3’
Human ACTIN	sense 5’ AGC-ACT-GTG-TTG-GCG-TAC-AG 3’<br>antisense 5’ GGA-CTT-CGA-GCA-AGA-GAT-GG 3’

Supplementary Table 2. ChIP primer sequences

Promoter	**sequence**
Nr5a2	sense 5’ TTC-AAG-TGG-GAG-GGG-TGG-AA 3’<br>antisense 5’ GCC-CGG-GAA-GAG-TCC-TAG-AT 3’
Arg 1	sense 5’ GCT-GTT-TTA-GCC-TCA-CCT-TGC 3’<br>antisense 5’ ATC-CGT-CCA-GTT-CAC-ACC-CT 3’
Cyp1a1	Sense 5’ CAT-CCC-CTG-GTT-CCC-TTG-GTA 3’<br>Antisense 5’ ACA-GAG-CAA-CAG-CAG-GGT-AT 3’
Cyp1b1	Sense 5’ CTT-TCC-TTG-GCC-ACT-GAT-CG 3’<br>Antisense 5’ TGG-ATG-GCA-CTC-TCT-CCA-TT 3’
Gapdh	Sense 5’ AGT-GCC-AGC-CTC-GTC-CCG-TAG-ACA-AAA-TG 3’<br>Antisense 5’ AAG-TGG-GCC-CCG-GCC-TTC-TCC-AT 3’
